# Analysis of Resistance of Ebola Virus Glycoprotein-Driven Entry Against MDL28170, An Inhibitor of Cysteine Cathepsins

**DOI:** 10.3390/pathogens8040192

**Published:** 2019-10-15

**Authors:** Markus Hoffmann, Svenja Victoria Kaufmann, Carina Fischer, Wiebke Maurer, Anna-Sophie Moldenhauer, Stefan Pöhlmann

**Affiliations:** 1 Infection Biology Unit, German Primate Center–Leibniz Institute for Primate Research, 37077 Göttingen, Germany; s.kaufmann02@stud.uni-goettingen.de (S.V.K.); carina.fischer01@stud.uni-goettingen.de (C.F.); wiebke.maurer@stud.uni-goettingen.de (W.M.); amoldenhauer@dpz.eu (A.-S.M.); 2 Faculty of Biology and Psychology, University Göttingen, 37073 Göttingen, Germany

**Keywords:** Ebola, cathepsin, glycoprotein

## Abstract

Ebola virus (EBOV) infection can cause severe and frequently fatal disease in human patients. The EBOV glycoprotein (GP) mediates viral entry into host cells. For this, GP depends on priming by the pH-dependent endolysosomal cysteine proteases cathepsin B (CatB) and, to a lesser degree, cathepsin L (CatL), at least in most cell culture systems. However, there is limited information on whether and how EBOV-GP can acquire resistance to CatB/L inhibitors. Here, we addressed this question using replication-competent vesicular stomatitis virus bearing EBOV-GP. Five passages of this virus in the presence of the CatB/CatL inhibitor MDL28170 were sufficient to select resistant viral variants and sequencing revealed that all GP sequences contained a V37A mutation, which, in the context of native GP, is located in the base of the GP surface unit. In addition, some GP sequences harbored mutation S195R in the receptor-binding domain. Finally, mutational analysis demonstrated that V37A but not S195R conferred resistance against MDL28170 and other CatB/CatL inhibitors. Collectively, a single amino acid substitution in GP is sufficient to confer resistance against CatB/CatL inhibitors, suggesting that usage of CatB/CatL inhibitors for antiviral therapy may rapidly select for resistant viral variants.

## 1. Introduction

The Ebola virus (EBOV), a member of the family *Filoviridae*, is an enveloped single-stranded RNA virus that causes a severe and frequently fatal disease, Ebola virus disease (EVD), in humans [1]. The West African EVD epidemic [2] and the ongoing EVD outbreak in the Democratic Republic of Congo [3] demonstrate that EBOV poses a severe threat to human health. This threat is compounded by recent findings demonstrating that EBOV can persist in convalescent patients who can relapse even months after having recovered from EVD and may transmit the virus to others [4,5,6,7]. At present, neither vaccines nor therapeutics have been approved by regulatory agencies for use in humans, although clinical trials indicate that a vaccine based on replication-competent vesicular stomatitis virus (VSV) bearing the EBOV glycoprotein (GP) can efficiently protect against EVD [8,9]. Therefore, new antiviral strategies need to be established and the first step in the viral replication cycle, viral entry into target cells, is an attractive target.

The viral GP is incorporated into the viral envelope and mediates binding of EBOV to target cells [10], mainly macrophages and dendritic cells [11]. Moreover, GP facilitates fusion of the viral envelope with the limiting membrane of endolysosomes [12], allowing delivery of the viral genome into the host cell cytoplasm. Binding and membrane fusion are carried out by different subunits of GP. The N-terminal surface unit, GP1, binds to host cell receptors via a receptor-binding domain (RBD), while the C-terminal transmembrane unit, GP2, harbors the functional elements required for membrane fusion, including a fusion peptide and heptad repeats [10]. Although GP1 can bind to attachment factors on the cell surface [13,14], only the interaction with the intracellular receptor Niemann-Pick C1 (NPC1) within host cell endolysosomes is essential for EBOV entry [13,14,15,16]. This interaction critically depends on previous processing of EBOV-GP by the endolysosomal, pH-dependent cysteine proteases cathepsin B (CatB) and cathepsin L (CatL) [15,16,17,18]. Processing removes a glycan cap and a mucin-like domain (MLD) from GP1 and results in a primed form of GP. Primed GP still contains the RBD linked to GP2 via a disulfide bond [19,20] and can fuse the viral membrane with an endolysosomal membrane upon receipt of a trigger. However, the nature of this trigger remains to be identified [21,22,23,24].

In the light of the important role of CatB/CatL in EBOV-GP-driven entry, inhibitors targeting these enzymes might hold therapeutic potential, although it should be stated that CatB/CatL dependence varies between filoviruses [25,26] and target cell types [27] and was not observed in experimentally infected mice [28]. A previous study employed a replication-competent VSV chimera bearing EBOV-GP without MLD (VSV-EBOV) to examine resistance development against the CatB inhibitor CA074 [29]. Resistance development was observed after two passages and was due to acquisition of single amino acid substitutions at the GP1-GP2 interface which increased susceptibility of the mutant GP to processing by host cell proteases [29]. In contrast, resistance development in the context of full-length EBOV-GP and other CatB/CatL inhibitors has not been examined.

Here, we show that passaging of VSV-EBOV in the presence of the CatB/CatL inhibitor MDL28170 results in rapid acquisition of a single amino acid exchange, V37A, in GP1, which confers resistance against MDL28170 and other CatB/CatL inhibitors.

## 2. Results

**Figure 1:**
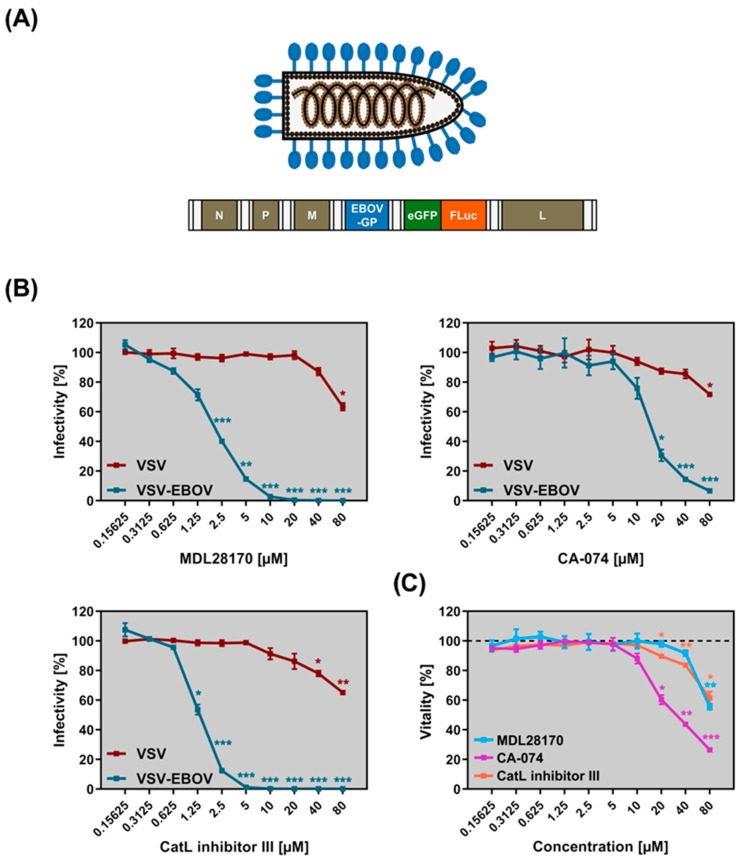
(**A**) Schematic illustration of the recombinant, replication-competent vesicular stomatitis virus (VSV) expressing the Ebola virus glycoprotein (EBOV-GP) instead of the parental VSV glycoprotein (VSV-G), VSV-EBOV, that was employed for the in vitro evolution experiment. The viral genome codes for a hybrid reporter consisting of an enhanced green fluorescent protein (eGFP) and firefly luciferase (FLuc) from an additional transcription unit. Abbreviations: N = nucleoprotein, P = phosphoprotein, M = matrix protein, L = polymerase. (**B**) Vero E6 cells were preincubated with cathepsin inhibitor (MDL28170, CA-074 or CatL inhibitor III) before being inoculated with replication-competent, recombinant VSV expressing either VSV-G (VSV) or EBOV-GP (VSV-EBOV) at a multiplicity of infection of 0.01. At 16 h postinfection, the activity of virus-encoded luciferase was measured in cell lysates. Shown are the combined data of three independent experiments for which the infectivity for cells treated with DMSO instead of inhibitor (control) was set as 100%. Error bars indicate SEM. Statistical significance of differences in the infection of inhibitor- versus control-treated cells was analyzed by one-way analysis of variance with Dunnett’s posttest (*p* > 0.05, no significant [not indicated]; *, *p* ≤ 0.05; **, *p* ≤ 0.01; ***, *p* ≤ 0.001). Inhibitory concentration 50 (IC50) values: 2.2 µM (MDL28170), 16.1 µM (CA-074), 1.3 µM (CatL inhibitor III). (**C**) Vero E6 cells were incubated for 24 h in the presence of the indicated concentrations of protease inhibitors MDL28170 [light blue], CA-074 [purple], and CatL inhibitor III [orange]) or DMSO (control) before cell viability was quantified. Presented are the combined data of three independent experiments for which the viability of control-treated cells was set as 100%. Error bars indicate SEM. Statistical significance of differences in cell viability between control- and inhibitor-treated cells was analyzed by one-way analysis of variance with Dunnett’s posttest (*p* > 0.05, not significant [not indicated]; *, *p* ≤ 0.05; **, *p* ≤ 0.01; ***, *p* ≤ 0.001).

In order to study resistance development against CatB/CatL inhibitors, we used a VSV-EBOV chimeric virus encoding firefly luciferase and eGFP (Figure 1A) and examined inhibition by the CatB and/or CatL inhibitors MDL28170, CA-074, and CatL inhibitor III. CA-074 is largely specific for CatB while MDL28170 and CatL inhibitor III inhibit both CatB and CatL [26,30,31]. Vero E6 cells were chosen for experimentation since this cell line allows for efficient replication of EBOV and has previously been used to study inhibition of EBOV-GP-driven entry by CatB/CatL inhibitors and the development of viral resistance against these inhibitors [17,29,32,33]. Preincubation of Vero E6 cells with these inhibitors followed by infection with VSV-EBOV universally resulted in efficient and dose-dependent inhibition of infection, with IC50 values of 1.3 µM (CatL inhibitor III), 2.2 µM (MDL28170), 16.1 µM (CA-074) (Figure 1B). Moreover, MDL28170 and CatL inhibitor III reduced VSV-EBOV infection to background levels when applied at 10 µM without eliciting unwanted cytotoxicity (Figure 1B,C). In contrast, 20 µM of CA-074 reduced VSV-EBOV infection by only 70% and caused measurable cytotoxic effects, although it should be stated that VSV infection was not affected at this concentration (Figure 1B,C). The lack of unspecific VSV inhibition despite measureable cytotoxicity might result from differences between incubation periods: Infection efficiency was measured at 18 h after addition of compound while cytotoxicity was measured at 24 h after addition of compound. In sum, we defined experimental conditions under which EBOV-GP- but not VSV-G-driven entry was blocked by CatB/CatL inhibitors in a concentration-dependent manner.

**Figure 2:**
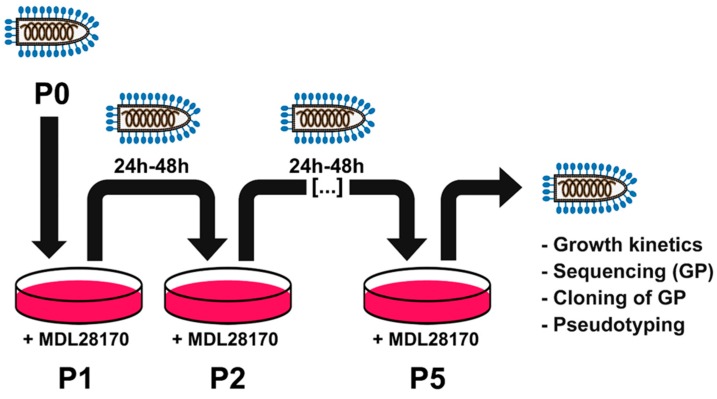
Set-up of the in vitro evolution experiment. Vero E6 cells were pretreated with cathepsin inhibitor MDL28170 before being inoculated with VSV-EBOV (= passage 0, P0). Supernatants were collected after 24–48 h (P1), cleared from cellular debris, diluted 1:100, and inoculated onto fresh, inhibitor-treated target cells (P2). This routine was repeated for a total of five passages (P3–P5), before supernatants were further analyzed.

MDL28170 was selected for further analysis because this compound caused a dose-dependent inhibition of VSV-EBOV over a broader range of concentrations (1.25–40 µM) than CA-074 (10–80 µM) or CatL inhibitor III (1.25–10 µM), while it did not have any non-specific side-effects on cell viability and infectivity of VSV up to a concentration of 40 µM. In order to determine how EBOV-GP can acquire resistance against MDL28170 the experimental setup depicted in Figure 2 was chosen. First, VSV-EBOV was passaged 5 times in Vero E6 cells in the presence of 20 µM MDL28170, the concentration that reduced VSV-EBOV infection by ~99.5% without causing unwanted cytotoxic effects. Then, MDL28170 sensitivity of passaged and control virus was analyzed and the GP sequences were determined (Figure 2). 

**Figure 3:**
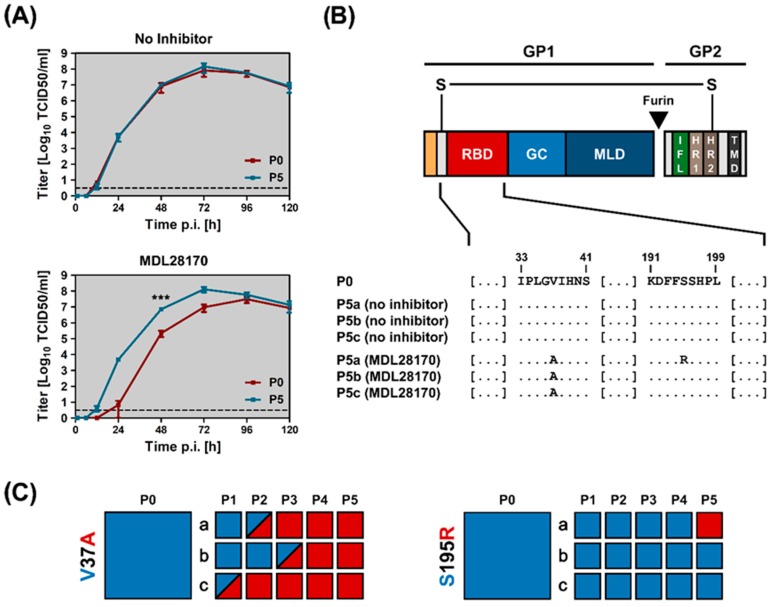
In vitro evolution affords VSV-EBOV P5 with a growth advantage in the presence of cathepsin inhibitor. (**A**) Unpassaged (P0, red) and passage 5 (P5, blue) VSV-EBOV were inoculated onto untreated (upper panel) or MDL28170-treated (20 µM, lower panel) Vero E6 cells at a multiplicity of infection of 0.005. Virus adsorption was allowed for 1 h. Next, the inoculum was removed and the cells were washed before fresh medium was added (for cells pretreated with cathepsin inhibitor, the medium was again supplemented with 20 µM MDL28170). Samples were taken at 1, 6, 12, 24, 48, 72, 96, and 120 h post infection. Viral titers were determined by inoculation of fresh Vero E6 cells with 10-fold serial dilutions of the supernatants (quadruplicates) and calculation of the tissue culture infectious dose 50 per ml (TCID 50/ml). Shown are the mean titers of three independent experiments. Error bars indicate SEM. Statistical significance of differences in the titers of P0 versus P5 virus in the presence of no inhibitor or MDL28170 was analyzed by two-way analysis of variance with Sidak’s posttest (*p* > 0.05, not significant [not indicated]; ***, *p* ≤ 0.001). (**B**) Schematic illustration of EBOV-GP. The two subunits, GP1 and GP2, are linked via a disulfide bond. The GP1 subunit harbors the signal peptide (orange), receptor-binding domain (RBD, red), glycan cap (GC, light blue), and mucin-like domain (MLD, dark blue). In addition, the GP2 subunit contains the internal fusion loop (IFL, green), two heptad repeat domains (HR1, light brown and HR2, dark brown) and the transmembrane domain (TMD, dark grey). Highlighted below the scheme are the regions in EBOV-GP, in which amino acid substitutions were detected within this study. The amino acid sequence of EBOV-GP from P0 VSV-EBOV is shown in the first row, followed by the corresponding sequences obtained after five passages in untreated (no inhibitor, control) or cathepsin inhibitor-treated (MDL28170) Vero E6 cells (dots indicate identical amino acid residues compared to P0 and the letters “a”, “b”, and “c” refer to independent experiments. (**C**) Chronological occurrence of V37A and S195R mutations during passaging. Each row of rectangles (a–c) represents independent experiments. Blue and red coloring highlights wildtype and mutant amino acid residues (rectangles with split colors indicate passages with mixed populations).

In the absence of MDL28170, control virus (passage 0, P0) and passage 5 virus replicated with comparable kinetics in control-treated cells (Figure 3A). In contrast, passage 5 virus replicated more efficiently in the presence of MDL28170 as compared to WT virus (Figure 3A), indicating that passaging was associated with MDL28170 resistance, most likely due to the acquisition of mutations. Indeed, sequencing revealed that passaging was invariably associated with emergence of amino acid substitution V37A and in some cases also S195R (Figure 3B,C). 

**Figure 4:**
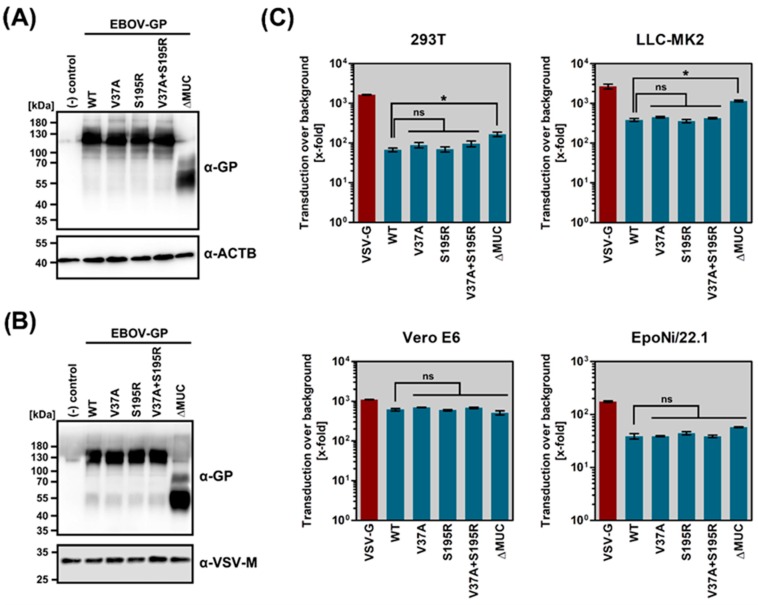
V37A does not affect EBOV-GP expression, particle incorporation, or host tropism. (**A**) 293T cells were transfected with expression plasmids coding for EBOV-GP WT, EBOV-GP V37A, EBOV-GP S195R, EBOV-GP V37A + S195R, EBOV-GP ∆MUC, or empty expression vector (negative control). At 48 h post transfection, whole cell lysates were prepared and subjected to SDS-PAGE and Western blotting. EBOV-GP was detected via incubation with a polyclonal rabbit serum directed against GP1 and a horseradish peroxidase-coupled anti-rabbit antibody. As a loading control, beta-actin (ACTB) was detected using anti-ACTB (mouse) and horseradish peroxidase-coupled anti-mouse antibodies. Presented are the data from one representative experiment that were confirmed in a separate experiment. Numbers on the left indicate the molecular weight in kilodalton (kDa). (**B**) Rhabdoviral vectors harboring no (negative control) or the indicated glycoprotein were pelleted via centrifugation through a 20% sucrose cushion and subjected to SDS-PAGE and Western blotting. EBOV-GP was detected as described for panel A. As a loading control, VSV-M was detected using anti-VSV-M (mouse) and horseradish peroxidase-coupled anti-mouse antibodies. Presented are the data from one representative experiment that were confirmed in a separate experiment. Numbers on the left indicate the molecular weight in kDa. (**C**) Rhabdoviral vectors harboring the indicated glycoproteins were inoculated onto 293T (human), Vero E6 (African green monkey), LLC-MK2 (rhesus macaque), or EpoNi/22.1 (fruit bat) cells. At 16 h post transduction, the activity of virus-encoded luciferase was measured in cell lysates. The combined results of three independent experiments are shown. Transduction was normalized against control particles bearing no glycoprotein (set as 1) and is shown as x-fold change over background. Error bars indicate SEM. Statistical significance of differences in transduction efficiency mediated by WT versus mutant EBOV-GP was tested by one-way analysis of variance with Dunnett’s posttest (*p* > 0.05, not significant [ns]; *, *p* ≤ 0.05).

The introduction of V37A and S195R into EBOV-GP did not interfere with robust GP expression in transfected cells (Figure 4A) and incorporation into VSV particles (Figure 4B). Moreover, both mutations did not alter EBOV-GP-driven entry into cell lines of fruit bat (natural reservoir, EpoNi/22.1), macaque (animal model, LLC-MK2), African green monkey (animal model, Vero E6), and human (accidental host, 293T) origin (Figure 4C). 

**Figure 5:**
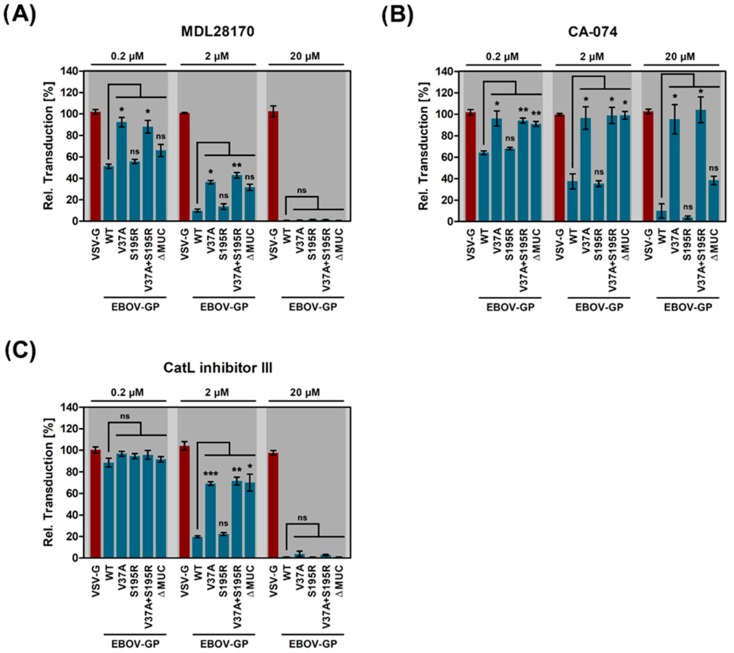
V37A reduces sensitivity of EBOV-GP-driven host cell entry to limited cathepsin availability. Rhabdoviral vectors harboring the indicated glycoproteins were inoculated onto Vero E6 cells pre-treated with different concentrations of cathepsin inhibitor (**A**) MDL28170, (**B**) CA-074, (**C**) CatL inhibitor III. At 16 h post transduction, the activity of virus-encoded luciferase was measured in cell lysates. Shown are the combined data of three independent experiments for which the transduction efficiency in cells treated with DMSO instead of inhibitor (control) was set as 100%. Error bars indicate SEM. Statistical significance of differences in the transduction of inhibitor-treated cells by particles harboring WT versus mutant EBOV-GP was analyzed by two-way analysis of variance with Dunnett’s posttest (*p* > 0.05, not significant [ns]; *, *p* ≤ 0.05; **, *p* ≤ 0.01; ***, *p* ≤ 0.001).

V37A conferred resistance against MDL28170, CA-074, and CatL inhibitor III while S195R failed to confer or enhance resistance when tested alone or in combination with V37A, respectively (Figure 5). Thus, a single amino acid mutation, V37A, rapidly arises upon passaging of VSV-EBOV in the presence of MDL28170 and confers resistance against this and related inhibitors.

## 3. Discussion

Multiple compounds have been shown to interfere with different steps of the EBOV lifecycle [34], including inhibitors of cellular cysteine proteases [32,35,36,37,38,39]. However, none of these compounds has been approved by regulatory agencies for treatment of EVD. The reasons for the lack of approved antiviral drugs include (i) unknown antiviral efficacy in vivo (most data stem from in vitro experimentation), (ii) potential unwanted side-effects in patients, and (iii) potential resistance-development by the virus during treatment. Therefore, promising compounds and new targets for intervention should be characterized regarding the aforementioned points in order to identify new options for treatment of EVD.

A previous study by Wong and colleagues demonstrated that passaging of VSV-EBOV lacking the MLD in the presence of a CatB inhibitor rapidly selected for mutations in GP1 or GP2 that conferred resistance, i.e., allowed efficient infection of target cells in the presence of inhibitor [29]. The mutations selected in GP1 resulted in amino acid substitutions at positions 40, 42, 43, or 47 at the N-terminus of EBOV-GP [29]. In the context of the properly folded mature protein, the region containing these amino acid residues is located in the GP1 base, which interacts with GP2 [40]. Moreover, some of these amino acid substitutions increased the efficiency of proteolytic processing of precleaved GP and rendered particles bearing precleaved GP more susceptible to inactivation by proteolytic digestion [29]. Consequently, it has been proposed that some of the mutations conferred resistance to the CatB inhibitor by destabilizing the GP trimer, thereby rendering it more accessible to proteolytic digestion by CatB/CatL and increasing the efficiency of GP triggering [29]. In addition, it has been postulated that some mutations render GP-driven entry CatB-independent by allowing GP processing by so far unidentified endosomal cysteine proteases other than CatB [29]. 

The present study confirms and extends the findings reported by Wong and coworkers: We show that also in the context of full-length EBOV-GP, a single amino acid substitution, V37A, located within the GP1 base, is rapidly acquired upon passaging of the virus in the presence of the inhibitor and is sufficient to confer resistance. In light of the almost identical location of this resistance mutation as compared to the ones described above [29], it is likely that they confer resistance via similar mechanisms. Moreover, we demonstrate that resistance development occurs in the presence of an inhibitor, MDL28170, that, unlike the CatB-specific compound CA-074 used in the previous study, blocks both CatB and CatL activity [30,31]. In this context, it should be mentioned that V37A conferred complete resistance against the largely CatB-specific inhibitor CA-074 (up to the highest concentration tested, 20 µM) but not the CatB/CatL-specific inhibitors MDL28170 and CatL inhibitor III, suggesting that the mutation might render entry CatB but not CatL independent. Thus, mutation V37A might induce changes in the EBOV-GP structure that render the CatB cleavage site also accessible for CatL or related cysteine proteases susceptible to inhibition by MDL28170 and CatL inhibitor III but not CA-074. Alternatively, V37A might destabilize GP in such a way that incomplete processing of GP by CatL and/or other proteases might be sufficient to render GP susceptible to the membrane fusion trigger.

Inhibitors blocking CatB and/or CatL activity and entry of EBOV, emerging coronaviruses and Nipah virus, which also rely on cysteine cathepsins for glycoprotein processing [31,41,42,43], have been described [37,38]. Moreover, they have been shown to block coronavirus spread and pathogenesis in rodent models [38]. Whether they will also block EBOV spread in the host and whether this will result in rapid emergence of resistant viruses as suggested by the present study remains to be determined. Finally, it will be interesting to investigate whether resistance comes at a cost—for instance, increased sensitivity towards antibody-mediated neutralization and/or augmented sensitivity towards blockade by interferon-induced antiviral effector proteins.

## 4. Materials and Methods 

### 4.1. Cell Culture

293T (human embryonic kidney; ATCC CRL-3216), Vero E6 (African green monkey kidney; ATCC CRL-1586), LLC-MK2 (rhesus macaque kidney; ATCC CCL-7) and EpoNi/22.1 (Buettikofer’s epauletted fruit bat kidney, [44], kindly provided by M.A. Müller and C. Drosten) cell lines were obtained from collaborators. The cells were grown in Dulbecco’s modified Eagle’s medium (DMEM; PAN Biotech) supplemented with 10% fetal bovine serum (FBS; PAN Biotech) as well as 1x penicillin and streptomycin from a 100x stock solution (PAN Biotech). The cell lines were cultivated at 37 °C and 5% CO_2_ in humidified atmosphere. Transfection of 293T cells was achieved by calcium phosphate precipitation.

### 4.2. Viruses

A recombinant VSV was employed that codes for a dual reporter consisting of enhanced green fluorescent protein (eGFP) and firefly luciferase (FLuc). This dual reporter is expressed from an additional expression cassette inserted between the open reading frames for VSV-G and VSV-L [45]. Further, a chimeric VSV was used that contains the genetic information for EBOV-GP (Mayinga variant, GenBank: AF086833.2) instead of the VSV glycoprotein (VSV-G) and thus leads to the production of VSV harboring EBOV-GP as the sole viral surface glycoprotein (= VSV-EBOV, Figure 1A) [45]. All viruses were propagated and titrated on Vero E6 cells.

### 4.3. Plasmids

We utilized previously described, pCAGGS-based expression plasmids for EBOV-GP WT, EBOV-GP∆MUC, a variant that lacks the mucin-like domain (amino acid residues 309–486), and VSV-G [46,47]. Empty pCAGGS vector served as negative control. Expression plasmids for mutant EBOV-GP harboring amino acid substitutions V37A, S195R, or V37A + S195R were constructed by overlap extension PCR using the plasmid for EBOV-GP WT as template. Sequence identity was confirmed by automated sequence analysis (Microsynth SeqLab).

### 4.4. Treatment of Cells with Cathepsin Inhibitors

In order to block activity of CatB and/or CatL, the following inhibitors were used: MDL28170 (calpain inhibitor that also inhibits CatB/L [26,30,31], Sigma-Aldrich), CA-074 (specific CatB inhibitor [26], Merck), and Cat L inhibitor III (CatL inhibitor that is also active against CatB [26], Merck). All inhibitors were reconstituted in dimethyl sulfoxide (DMSO) and prepared as stock solutions of 50 mM concentration. Target cells were treated with inhibitor 2 h before infection/transduction and cells treated with DMSO instead of inhibitor served as control. In order to ensure long-lasting CatB/L inhibition during the in vitro-evolution experiment the medium added to the cells after washing away the viral inoculum was also supplemented with inhibitor (or DMSO, control).

### 4.5. Infection of Target Cells with VSV-EBOV

For identification of the optimal inhibitor concentration for subsequent in vitro-evolution experiments, target cells pre-treated with DMSO (control) or increasing concentrations of inhibitor were inoculated with either VSV or VSV-EBOV using a multiplicity of infection (MOI) of 0.1. In case of in vitro-evolution experiments, VSV-EBOV was used at an MOI of 0.005. For infection, the cell culture supernatants containing DMSO/inhibitor were removed and cells were inoculated with the virus for 1 h at 37 °C and 5% CO_2_. Afterwards, the inoculum was removed and the cells were washed with PBS, before medium containing fresh DMSO/inhibitor was added. In case of experiments addressing the optimal inhibitor concentration, the infection was stopped at 16 h post-infection. For in vitro-evolution experiments, the supernatants were harvested when >90% of the cells were positive for eGFP expression (judged by fluorescence microscopy), which was typically achieved after 24–48 h. The harvested supernatants were freed from cellular debris by centrifugation, aliquoted and stored at −80 °C. For the consecutive inoculation of fresh target cells, an aliquot of the respective previous passage was thawed and diluted 1:100 for inoculation.

### 4.6. Quantification of Viral Infection

To identify the optimal inhibitor concentration for subsequent in vitro-evolution experiments, replication of VSV and VSV-EBOV was analyzed by measuring FLuc activity in cell lysates at 16 h post-infection using the Beetle juice kit (PJK) and a plate luminometer (Hidex). For normalization, infection efficiency (indicated by FLuc activity) for DMSO-treated cells was set as 100% and the relative infection efficiencies for cells pre-treated with increasing concentrations of inhibitor were calculated accordingly. In order to compare viral growth kinetics of unpassaged VSV-EBOV and VSV-EBOV after 5 passages in inhibitor-treated cells, viral titers were determined by calculating the tissue culture infectious dose 50 per ml (TCID50/ml) as described before [45].

### 4.7. Preparation of Rhabdoviral Transduction Vectors and Transduction of Target Cells

We employed rhabdoviral transduction vectors that are based on a replication-deficient VSV coding for eGFP and FLuc instead of the VSV-G (kindly provided by G. Zimmer, [48]). Pseudotyping of this vector with WT or mutant EBOV-GP, or VSV-G (positive control) was performed according to a published protocol [49]. Empty particles produced in the absence of any viral glycoprotein served as negative control. For transduction, target cells were inoculated with equal volumes of supernatants containing pseudotyped VSV. At 16 h post-transduction, the efficiency of transduction was quantified by measuring FLuc activity in cell lysates according to published protocol [49].

### 4.8. Western Blot Analysis

Total expression of WT and mutant EBOV-GP as well as particle incorporation was investigated by SDS-PAGE (sodium polyacrylamide gel electrophoresis) and Western blot analysis. Whole cell lysates (WCL) of cells expressing WT or mutant GP (or no GP, negative control) were prepared as follows: At 48 h post-transfection, culture supernatants were removed. Then, the cells were washed with PBS and mixed with 2x SDS sample buffer (0.03 M Tris-HCl, 10% glycerol, 2% SDS, 0.2% bromophenol blue, 5% beta-mercaptoethanol, 1 mM EDTA) for cell lysis. After 10 min of incubation at room temperature, samples were further heated to 96 °C for an additional 10 min. To analyze EBOV-GP particle incorporation, rhabdoviral transduction vectors were concentrated via centrifugation through a 20% sucrose cushion (17,000× *g*, 2 h, 4 °C) before being mixed with 2× SDS sample buffer and incubated as described above.

After SDS-PAGE, the proteins were blotted onto nitrocellulose membranes (Hartenstein GmbH), which were further blocked by incubation in PBS-T (PBS containing 0.5% Tween 20 and 5% skim milk powder) for 30 min at room temperature. Incubation with the primary antibody (diluted in PBS-T) was performed overnight at 4 °C. For detection of EBOV-GP, we used a GP1-specific serum (rabbit, 1:2500, [50]), while for detection of the loading controls beta-actin (ACTB, WCL) and VSV matrix protein (VSV-M, particle incorporation), anti-ACTB (mouse, 1:1000, Sigma-Aldrich) and anti-VSV-M antibodies (mouse, 1:1000, Kerafast) were used, respectively. Next, the membranes were washed three times with PBS-T before incubation with the horseradish (HRP)-conjugated secondary antibodies, anti-rabbit-HRP (goat, 1:5000, Dianova) or anti-mouse-HRP (goat, 1:5000, Dianova), was performed for 1 h at room temperature. After an additional wash step, the membranes were incubated with an in house-prepared enhanced chemiluminescent solution (0.1 M Tris-HCl [pH 8.6], 250 µg/ml luminol, 1 mg/ml para-hydroxycoumaric acid, 0.3% H_2_O_2_) and signals were recorded using the ChemoCam imaging system and the ChemoStar Professional software (Intas Science Imaging Instruments GmbH).

### 4.9. Analysis of Cytotoxicity

Unwanted cytotoxic effects of protease inhibitor treatment were analyzed using the CellTiter-Glo assay (Promega), following the manufacturer’s instructions. In brief, Vero E6 cells were seeded in 96-well plates and incubated with inhibitor for 24 h. Subsequently, assay substrate was added and luminescence quantified using a plate luminometer (Hidex).

### 4.10. Statistical Analysis

We performed one- and two-way analysis of variance (ANOVA) with either Dunnet’s (comparison of multiple samples with a single reference sample) or Sidak’s posttest (multi-step comparison of two samples over time) using the GraphPad Prism 7 software package (GraphPad Software). Only *p* values of 0.05 or lower were considered statistically significant (*p* > 0.05 [ns, not significant], *p* ≤ 0.05 [*], *p* ≤ 0.01 [**], *p* ≤ 0.005 [***]).
